# Evaluation of Spiritual Care and Well-Being Levels of Individuals Diagnosed with Lung Cancer in Turkey

**DOI:** 10.1007/s10943-024-02088-3

**Published:** 2024-08-23

**Authors:** Seher Çakmak, Melike Demir Doğan, Nisanur Selim, Gülse Nur Kalleci

**Affiliations:** 1 Department of Internal Medicine Nursing, Faculty of Health Sciences, Gümüşhane University, Gümüşhanevî Campus, Bağlarbaşı Street, 29100 Gümüşhane, Turkey; 2 Department of Nursing, Faculty of Health Sciences, Gümüşhane University, Gümüşhanevî Campus, Bağlarbaşı Street, 29100 Gümüşhane, Turkey

**Keywords:** Lung cancer, Spirituality, Spiritual well-being, Spiritual care

## Abstract

This study aimed to assess the spiritual care needs and spiritual well-being levels of lung cancer patients undergoing chemotherapy (CT). This descriptive cross-sectional study was conducted with 110 patients in the outpatient CT unit of a university hospital. Data were collected using a personal information form, the “Three-Factor Spiritual Well-Being Scale” and the “Spiritual Care Needs Scale.” The average age of participants was 62.6 ± 8.0 years. Patients with a university or above education level, civil servants, self-employed individuals, those receiving only CT, and those with less than 5 CT cycles had significantly higher spiritual well-being scores (*p* < 0.05). Spiritual care needs scale scores were significantly higher for married individuals and those receiving only CT (*p* < 0.05). In conclusion, both spiritual well-being levels and spiritual care needs were observed to be high among lung cancer patients.

## Introduction

Lung cancer, a prevalent malignancy globally and in our country, predominantly affects men. Individuals with lung cancer endure various symptoms arising from both the disease and its treatment (Andersen et al., 2020). These symptoms include physical manifestations like fatigue, anorexia, dyspnea, cough, pain, and hemoptysis, alongside psychological distress like irritability, sleep disturbances, sadness, hopelessness, depression, and anxiety. These symptoms profoundly impact patients’ emotional, social, and spiritual well-being, thus affecting their overall quality of life (Akin et al., 2010; Ellis, 2012).

### Spiritual Well-Being in Lung Cancer

Healthy living reflects a sense of well-being that results from an intense awareness of wholeness and integration between all dimensions of one's being, including the spiritual elements of life. Spiritual well-being, which is the combination of spirituality and health, is defined as a person establishing coordinated and harmonious relationships with God, himself and others, and producing fair and balanced solutions (Bożek et al., 2020). According to another definition, spiritual well-being refers to a lifestyle that aims to improve a person’s mental, physical and social functioning and shape the perception of health and illness. Spiritual well-being includes finding meaning in life, inner peace, self-acceptance, having a sense of personal growth, religiosity, and self-esteem. Spiritual well-being is a crucial aspect of quality of life and shapes individuals’ perceptions of health and illness. (Jetan et al., 2023). Three sub-dimensions of Spiritual Well-Being were defined by Ekşi and Kardaş (2017). These are transcendence—harmony with nature and anomie. Self-transcendence—the realization that one goes beyond oneself and is a part of the existing whole. Harmony—with nature is the individual’s preservation of the connection between his/her own life and her natural environment. Anomie means that social rules lose their power to direct and control people’s behavior, and people are indecisive about how to behave (Vieten et al., 2023).

For cancer patients, spiritual well-being serves as a vital reservoir of strength and coping mechanisms. Conversely, individuals with lower spiritual well-being often experience high physical and psychological distress (Lo et al., 2011; Meraviglia, 2004). In a study by Vallurupalli et al. (2012) involving advanced cancer patients, it was observed that a significant portion of participants relied on their religious or spiritual beliefs to navigate the challenges posed by cancer (Vallurupalli et al., 2012). This underscores the pivotal role of spirituality in coping with the complexities of cancer diagnosis and treatment.

In lung cancer patients, higher levels of spiritual well-being are associated with lower depressive symptoms and reduced cancer distress among patients (Cho et al., 2021). Various studies have indicated that high levels of spiritual well-being correlate with stronger religious beliefs, higher educational attainment, self-perceived better physical health, and lower anxiety levels, while lower spiritual well-being is linked to feelings of hopelessness, depression, anxiety, a lack of social support, diminished quality of life, and prolonged hospitalization (Hiratsuka et al., 2021; Vallurupalli et al., 2012).

### Spiritual Needs in Lung Cancer

Given that cancer is often perceived by patients as a life-threatening condition, their spiritual needs become significantly more pronounced. Commonly reported spiritual needs in cancer patients include seeking inner peace, nurturing hope, establishing connections or relationships with the world, finding meaning and purpose in life, and experiencing transcendence (Bussing et al., 2010; Ripamonti et al., 2018). Numerous studies have shown that a cancer diagnosis prompts individuals to contemplate the meaning of life and the nature of existential pain; spiritual needs are often intertwined with concerns about the loss of roles and self-identity, as well as the fear of death, and any patients attempt to make sense of life in relation to an unseen or sacred world (Ahmadi et al., 2022; Maiko et al., 2019; Vos, 2014).

In various studies, cancer patients have been observed to turn to spiritual practices, including praying, sacrificing, visiting shrines, and engaging in rituals such as drinking Zamzam, along with conventional medical treatments, in their quest for healing (Rizalar et al., 2023). Another study reported that spirituality became a focal point for cancer patients, offering them a positive perspective and bolstering hopes for recovery; however, negative consequences were noted, such as patients discontinuing treatment in favor of emphasizing spirituality (Zumstein-Shaha et al., 2020). Furthermore, the research highlighted that the most frequently expressed spiritual needs among cancer patients were positivity, gratitude, hope, and peace, while “morality and ethics” were the least frequently mentioned (Forouzi et al., 2017).

Spiritual needs have been found to exert a negative impact on mental health and increase psychological distress in lung cancer patients (Fradelos et al., 2021). Patients express a desire for healthcare professionals, including doctors and nurses, to consider and address their spiritual needs as part of cancer care (Vallurupalli et al., 2012). In a study, it was revealed that nearly all patients with advanced cancer had spiritual needs and sought spiritual care from healthcare professionals, clergy, and religious communities (Pearce et al., 2012). When patients’ spiritual needs are met, it positively influences their quality of life. However, the study suggests that the spiritual needs of cancer patients are not adequately supported by religious communities or the medical system (Balboni et al., 2011).

Cancer patients whose spiritual needs are not met may experience many spiritual distress such as refusal of care, fear of being alone, introversion, depression, anxiety, social isolation, and decreased self-confidence (Pearce et al., 2012). Identifying and addressing spiritual needs may increase the spiritual well-being levels of patients. Although there are studies examining the spiritual well-being levels or spiritual care needs of lung cancer patients, there is a paucity of the literature evaluating the spiritual well-being levels and spiritual care needs of these patients together (Fradelos et al., 2021; Kahraman & Pehlivan, 2023; Kırca et al., 2023).

This study aims to determine the spiritual well-being levels and spiritual care needs of lung cancer patients receiving outpatient treatment. It is thought that this study will contribute to the understanding of spiritual well-being levels and spiritual care needs among lung cancer patients, identify factors influencing their spiritual well-being, and provide insights for healthcare professionals, especially nurses, to recognize and address spiritual care needs as an integral part of nursing care.

### Research Questions

1. What is the average spiritual well-being score among the patients?
2. What factors influence the spiritual well-being of these patients?
3. What is the average score for the spiritual care needs of the patients?
4. What factors affect the spiritual care needs of these patients?

## Methods

### Study Design

This study is a descriptive and cross-sectional study.

### Sample and Setting

The study, conducted between May and October 2023 at the outpatient chemotherapy unit of Karadeniz Technical University (KTU) Application and Research Center (Farabi Hospital), aimed to explore the spiritual well-being levels and spiritual care needs of lung cancer patients who had undergone chemotherapy in the past year. The sample size, determined using a 95% confidence interval and a 0.05 margin of error, was calculated as 110 individuals using a known population formula ("https://www.openepi.com/SampleSize/SSCohort.htm"). Participants were selected through the random sampling method, ensuring representation from the population of interest. Inclusion criteria encompassed voluntary agreement to participate, age 18 and above, and the absence of communication difficulties.

### Data Collection Tools

The data were collected using three instruments: The “Personal Information Form,” the “Three-Factor Spiritual Well-Being Scale (TFSWBS),” and the “Spiritual Care Needs Scale (SCNS).”

### The Personal Information Form

The Personal Information Form was designed by the researchers after a literature review (Cho et al., 2021; Martins et al., 2020), comprising 15 questions related to patients’ sociodemographic characteristics (age, gender, education level, etc.) and aspects regarding their disease and treatment (diagnosis, diagnosis time, disease status, treatment received, treatment protocol, etc.).

### The Three-Factor Spiritual Well-Being Scale (TFSWBS)

The “Three-Factor Spiritual Well-Being Scale,” originally developed as the “Spiritual Well-Being Scale” by Ekşi and Kardaş in 2017 (Ekşi & Kardaş, 2017), underwent a name change to the “Three-Factor Spiritual Well-Being Scale” in 2019 to prevent confusion with a different scale developed by Paloutzian and Ellison (1982) (Kardaş, 2019; Paloutzian & Ellison, 1982). Comprising 29 items, the scale assesses three subdimensions: transcendence, harmony with nature, and anomie. Self-transcendence is the realization that one goes beyond oneself and is a part of the existing whole. Harmony with nature is the individual's preservation of the connection between his/her own life and her natural environment. Anomie means that social rules lose their power to direct and control people's behavior, and people are indecisive about how to behave. The subdimension scores are calculated by summing specific items: items 1, 4, 5, 8, 9, 12, 13, 16, 17, 20, 21, 24, 25, 27, and 29 for the transcendence subdimension; items 2, 6, 10, 14, 18, 22, and 28 for the harmony with nature subdimension; and items 3, 7, 11, 15, 19, 23, and 26 for the anomie subdimension. Higher scores in each subdimension signify a higher manifestation of the evaluated characteristic. The scale also provides a total well-being score, calculated as the average of the subdimensions, with reverse scoring applied to items in the anomie subdimension. In the validity and reliability study, the Cronbach Alpha value of the scale was found to be 0.953 in the “transcendence” subdimension, 0.864 in the “harmony with nature” subdimension, 0.853 in the “anomie” subdimension and 0.886 in the total score (Ekşi & Kardaş, 2017). The Cronbach Alpha values were found to be 0.962 for the “transcendence” subdimension, 0.890 for “harmony with nature,” 0.773 for “anomie,” and 0.931 for the total score in this study.

### The Spiritual Care Needs Scale

Otuzoğlu (2017) devised the scale for assessing the “spiritual care needs of oncology patients”. Originally planned with 58 items, the trial scale underwent statistical evaluation and eventually settled on a final form comprising 24 items. Respondents use a 5-point Likert-type scale, selecting from options such as “not at all, only a little, to some extent, rather much, and very much”. The subdimensions of the scale are faith and spiritual practices, to be peaceful and secure until the end of life, love and support of relatives and informing them about their health. The scale yields a minimum score of 24 points and a maximum score of 120 points. Higher total scores indicate an increase in the spiritual care needs of the patients (Otuzoğlu, 2017).

### Data Collection Procedure

Data collection occurred after informing and obtaining verbal consent from the patients. Face-to-face interviews were conducted with lung cancer patients undergoing treatment in the outpatient CT unit, supplemented by patient file reviews. The researcher read out questions related to personal characteristics and scale items, recording patient responses. Patient files provided information on diagnosis and treatment. The questionnaire took, on average, 15–20 min to complete.

### Data Analyses

For data analysis, the IBM SPSS 21.0 statistical program was employed. Descriptive analyses (frequency, percentage, mean, standard deviation, median, and minimum–maximum values) were conducted. The Kolmogorov–Smirnov test was utilized to assess conformity to the normal distribution and comparison tests were performed between groups. In the comparison of scale scores for categorical data, the following statistical tests were employed: the *t* test for data conforming to normal distribution when comparing two independent groups; the Mann–Whitney U test for data not adhering to normal distribution; the one-way ANOVA test for data fitting normal distribution when comparing more than two independent groups; and the Kruskal–Wallis test for data not fitting normal distribution. To compare mean scores across more than two groups, the Bonferroni correction was applied to identify the group with a significant difference. The significance level was set at *p* < 0.05.

## Results

Table 1 presents the descriptive characteristics of the patients. The average age of the patients was 62.6 ± 8.0 years, with 63.6% being over 60 years old. 75.5% of the participants were male, 92.7% were married, 62.7% had completed primary school, 30.9% were retired, 88.2% had a moderately good income, 9.1% were employed, and 99.1% had health insurance (Table 1).

**Table 1:** Descriptive characteristics of the patients (*n* = 110)

Descriptive characteristics		N	%
Age			
Mean: 62.6 ± 8.0	60 ≤	40	36.4
Range 46–83	60 >	70	63.6
Gender	Female	27	24.5
	Male	83	75.5
Marital status	Married	102	92.7
	Single	8	7.3
Educational status	Illiterate	2	1.8
	Primary education	69	62.7
	High school	20	18.2
	University and over	19	17.3
Occupation	Housewife	17	15.5
	Retired	34	30.9
	Civil servant	19	17.3
	Self-employed	29	26.4
	Worker	11	10.0
Income status	Moderate	97	88.2
	High	13	11.8
Employment status	Yes	10	9.1
	No	100	90.9
Health insurance	Yes	109	99.1
	No	1	0.9

Table 2 shows the diagnostic and treatment characteristics of the patients. 95.4% of the patients had a diagnosis of lung CA, 52.7% had a diagnosis year between 0 and 1 year, 65.5% had primary disease, 39.1% had a history of surgery, 65.5% had a history of RT, 65.5% were currently receiving only CT, 20% were receiving Nivolumab CT, 29.1% were receiving more than 20 cycles of CT, and 49.1% were receiving CT every 21 days (Table 2).

**Table 2:** Diagnosis and treatment characteristics of the patients (*n* = 110)

Diagnosis and treatment characteristics		N	%
Diagnosis	Lung CA	105	95.4
	Bronchial or lung malignant neoplasm	2	1.8
	Small cell lung CA	3	2.7
Diagnosis time	0–1 years	58	52.7
	1–3 years	30	27.3
	3 years and over	22	20.0
Disease condition	Primary	72	65.5
	Metastatic	38	34.5
History of surgery	Yes	43	39.1
	No	67	60.9
History of radiotherapy	Yes	72	65.5
	No	38	34.5
Current treatment	Chemotherapy	72	65.5
	Chemo-radiotherapy	3	2.7
	Target treatment	35	31.8
Treatment protocol	Nivolumab	22	20.0
	Carboplatin/Paclitaxel	13	11.8
	Etoposide	12	10.9
	Cisplatin	9	8.2
	Carboplatin	7	6.4
	Pemetrexed	7	6.4
	Others*	40	36.4
Number of treatment courses	0–5. courses	31	28.2
	6–10. courses	23	20.9
	11–20. courses	24	21.8
	More than 20 courses	32	29.1
Treatment cycle	Weekly	15	13.6
	Every 15 days	38	34.5
	Every 21 days	54	49.1
	Every 28 days	3	2.7

^*^Atezolizumab, Bevacizumab / Irinotecan, Bevacizumab / Calcium Folinate, Calcium Folinate, Carboplatin / Etoposide, Carboplatin / Etoposide / Atezolizumab, Carboplatin / Gemcitabine, Carboplatin / Pemetrexed, Cetuximab / Fluorouracil / Calcium Folinate, Cisplatin / Pemetrexed, Cisplatin / Docetaxel, Etoposide / Atezolizumab, Gemcitabine, Gemcitabine/Cisplatin, Irinotecan / Calcium Folinate, Oxaliplatin, Paclitaxel, Paclitaxel / Carboplatin, Pembrolizumab, Pembrolizumab / Cisplatin, Rituximab, Vinorelbine / Cisplatin, Irinotecan, Zoledronic acid

The mean scores of the patients in the TFSWBS were found to be 65.7 ± 11.0 for the “transcendence” subdimension, 31.8 ± 3.9 for the “harmony with nature” subdimension, 24.9 ± 5.6 for the “anomie” subdimension and 118.6 ± 15.7 for the total score of the scale (Table 3).

**Table 3:** The mean scores of patients on the TFSWBS (n = 110)

Scale and subdimensions	Min–max	Mean ± SD	Median
Transcendence	23–75	65.7 ± 11.0	71.0
Harmony with nature	20–35	31.8 ± 3.9	33.0
Anomie	10–35	24.9 ± 5.6	25.0
TFSWBS	78–140	118.6 ± 15.7	125.0

*Min–max* Minimum–maximum, *SD* Standard deviation, *TFSWBS* Three-Factor Spiritual Well-Being Scale

Table 4 shows the comparison of mean scores on the TFSWBS subdimensions and total scores based on the descriptive characteristics of the patients. Statistical analysis revealed significant differences in the mean scores among different groups. Bonferroni correction was performed for significant differences between three or more groups. Specifically, the mean score for the subdimension of “harmony with nature” was found to be significantly higher in patients with university and above graduates than those with primary school education, and in patients who were civil servants than workers (*p* < 0.05). Moreover, the mean score for the subdimension of "anomie" was higher in patients with university and above graduates than in those with high school graduates. Additionally, the overall mean total score of the TFSWBS was significantly higher in patients with self-employment than workers (*p* < 0.05). On the other hand, no statistically significant difference was found between the mean scores of the subdimension and total scores of the TFSWBS according to the age, gender, marital status and employment status (*p* > 0.05) (Table 4).

**Table 4:** Comparison of the mean scores of the subdimension and total scores of the TFSWBS according to the descriptive characteristics of the patients (*n* = 110)

Descriptive characteristics		N (%)	Subdimensions and total mean scores			
			Transcendence X ± SD	Harmony with nature X ± SD	Anomie X ± SD	Total X ± SD
Age	60 ≤	40 (36.4)	65.4 ± 11.6	32.0 ± 3.6	24.4 ± 5.8	118.1 ± 15.3
	60 >	70 (63.6)	65.9 ± 10.7	31.6 ± 4.0	25.2 ± 5.5	118.9 ± 16.0
Test *p*			U = 1399.500 *p* = 0.998	U = 1379.000 *p* = 0.891	U = 1291.500 *p* = 0.499	U = 1345.000 *p* = 0.732
Gender	Female	27 (24.5)	66.3 ± 10.8	31.7 ± 3.1	25.0 ± 5.8	119.5 ± 13.6
	Male	83 (75.5)	65.5 ± 11.1	31.8 ± 4.1	24.8 ± 5.6	118.3 ± 16.4
Test *p*			U = 1109.500 *p* = 0.939	U = 998.000 *p* = 0.371	U = 1100.500 *p* = 0.889	U = 1098.500 *p* = 0.878
Marital status	Married	102 (92.7)	65.9 ± 11.0	31.8 ± 3.9	25.1 ± 5.6	119.0 ± 15.9
	Single	8 (7.3)	63.3 ± 12.2	31.5 ± 3.4	22.0 ± 5.7	113.6 ± 13.5
Test *p*			U = 313.500 *p* = 0.273	U = 355.000 *p* = 0.521	U = 257.000 *p* = 0.082	U = 287.500 *p* = 0.165
Education level	^1^Illiterate	2 (1.8)	73.0 ± 1.4	35.0 ± 0.0	32.0 ± 2.8	135.0 ± 4.2
	^2^Primary education	69 (62.7)	66.4 ± 9.1	31.1 ± 4.0	24.5 ± 5.6	118.3 ± 15.8
	^3^High school	20 (18.2)	67.7 ± 9.1	31.9 ± 4.0	22.3 ± 5.5	118.5 ± 14.1
	^4^University and over	19 (17.3)	60.4 ± 17.1	33.8 ± 2.2	28.0 ± 4.0	118.0 ± 17.7
Test *p*			KW = 2.631 *p* = 0.452	KW = 10.354 ***p* = 0.016** **4 > 2** *p = 0.004	*F* = 5.024 ***p* = 0.003** **4 > 3** *p = 0.002	*F* = 0.738 *p* = 0.532
Occupation	^1^Housewife	17 (15.5)	68.1 ± 8.0	31.8 ± 4.7	25.4 ± 6.5	121.3 ± 16.7
	^2^Retired	34 (30.9)	63.6 ± 12.6	31.0 ± 3.8	23.2 ± 4.5	114.3 ± 15.8
	^3^Civil servant	19 (17.3)	64.9 ± 13.7	33.4 ± 3.2	26.8 ± 4.8	121.1 ± 16.4
	^4^Self-employed	29 (26.4)	68.7 ± 8.8	32.7 ± 3.1	25.4 ± 6.0	123.2 ± 13.3
	^5^Worker	11 (10)	61.9 ± 8.4	29.1 ± 4.2	24.4 ± 6.8	111.5 ± 15.3
Test *p*			KW = 6.218 *p* = 0.183	KW = 12.474 ***p* = 0.014** **3 > 5** *p = 0.004	*F* = 1.494 *p* = 0.209	KW = 10.945 ***p* = 0.027** **4 > 5** *p = 0.013
Employment status	Yes	10 (9.1)	61.0 ± 14.5	31.9 ± 3.5	27.7 ± 6.1	116.7 ± 18.2
	No	100 (90.9)	66.2 ± 10.6	31.8 ± 3.9	24.6 ± 5.5	118.8 ± 15.5
Test *p*			*U* = 380.500 *p* = 0.211	*U* = 489.500 *p *= 0.909	*t* = − 1.690 *p* = 0.094	*U* = 484.500 *p *= 0.872

Bold values indicate statistical significance (*p* < 0.05)

^*^Mann–Whitney *U* Bonferroni correction

Table 5 outlines the comparison of mean scores on the TFSWBS subdimensions and total scores based on the diagnosis and treatment characteristics of the patients. Notably, the analysis revealed several statistically significant findings. Bonferroni correction was performed for significant differences between three or more groups. The mean score of the “transcendence” subdimension was significantly higher in patients receiving chemotherapy than in those receiving other treatments (*p* = 0.034). Moreover, patients with a diagnosis of 0–1 year demonstrated significantly higher mean scores on the “anomie” subdimension than the scores of those with a diagnosis of 3 years and more, and patients without a history of radiotherapy had significantly higher mean scores on the “anomie” subdimension than the scores of those with a history of radiotherapy (*p* = 0.006, *p* = 0.016, respectively). Furthermore, patients with 5 or fewer treatment cycles exhibited significantly higher mean scores on the “harmony with nature” and “anomie” subdimensions and total scores compared to those with more than 20 cycles (*p* = 0.004, *p* = 0.006, *p* = 0.009, respectively). On the other hand, no statistically significant difference was found between the mean scores of the subdimension and total scores of the TFSWBS according to the disease condition, history of surgery and treatment cycle (*p* > 0.05) (Table 5).

**Table 5:** Comparison of the mean scores of the subdimensions and total scores of TFSWBS based on the diagnostic and treatment characteristics of the patients (*n* = 110)

Diagnosis and treatment characteristics		N (%)	Subdimensions and total mean scores			
			Transcendence	Harmony with nature X ± SD	Anomie X ± SD	Total X ± SD
Diagnosis time	^1^0–1 years	58 (52.7)	65.2 ± 11.9	32.1 ± 4.0	25.9 ± 5.3	119.2 ± 16.7
	^2^1–3 years	30 (27.3)	64.9 ± 10.7	31.5 ± 3.6	24.7 ± 6.3	117.5 ± 14.8
	^3^3 years and over	22 (20)	68.0 ± 8.8	31.3 ± 3.9	22.4 ± 4.9	118.4 ± 14.6
Test *p*			*F* = 0.625 *p* = 0.537	*F* = 0.434 *p* = 0.649	*F* = 3.270 ***p* = 0.042** **1 > 3** *p = 0.006	*F* = 0.117 *p* = 0.889
Disease condition	Primary	72 (65.5)	65.9 ± 10.6	31.3 ± 3.8	24.4 ± 5.5	118 ± 15.7
	Metastatic	38 (34.5)	65.3 ± 11.8	32.6 ± 3.8	25.8 ± 5.7	119.7 ± 15.8
Test *p*			*t* = 0.253 *p* = 0.801	*t* = − 1.677 *p* = 0.096	*t* = − 1.296 *p* = 0.198	*t* = − 0.520 *p* = 0.604
History of surgery	Yes	43 (39.1)	66.7 ± 11.0	32.3 ± 3.6	25.3 ± 6.0	120.4 ± 15.4
	No	67 (60.9)	65.1 ± 11.1	31.4 ± 4.0	24.6 ± 5.4	117.5 ± 15.9
Test *p*			*t* = − 0.753 *p* = 0.453	*t* = − 1.279 *p* = 0.204	*t* = − 0.585 *p* = 0.560	*t* = − 0.943 *p* = 0.348
History of radiotherapy	Yes	72 (65.5)	65.2 ± 10.5	31.5 ± 3.7	23.9 ± 5.9	117.0 ± 15.6
	No	38 (34.5)	66.7 ± 11.9	32.3 ± 4.1	26.6 ± 4.6	121.6 ± 15.6
Test *p*			*t* = 0.692 *p* = 0.491	*t* = 1.040 *p* = 0.301	*t* = 2.454 ***p* = 0.016**	*t* = 1.449 *p* = 0.150
Current treatment	Chemotherapy	72 (65.5)	67.3 ± 10.3	32.2 ± 3.4	25.0 ± 5.6	120.7 ± 14.8
	Other (Chemo-radiotherapy or target therapy)	3 (2.7)	62.7 ± 11.7	31.0 ± 4.6	24.5 ± 5.8	114.7 ± 16.8
Test *p*			*t* = 2.147 ***p* = 0.034**	*t* = 1.465 *p* = 0.146	*t* = 0.456 *p* = 0.649	*t* = 1.915 *p* = 0.058
Number of treatment courses	^1^0–5. courses	31 (28.2)	67.1 ± 13.2	33.2 ± 3.1	26.3 ± 4.6	122.5 ± 15.5
	^2^6–10. courses	23 (20.9)	68.3 ± 8.1	32.8 ± 2.8	25.6 ± 5.2	122.9 ± 11.6
	^3^11–20. courses	24 (21.8)	65.3 ± 8.9	31.0 ± 4.2	25.7 ± 5.7	118.1 ± 14.7
	^4^More than 20 courses	32 (29.1)	62.8 ± 11.7	30.2 ± 4.3	22.3 ± 6.1	112.1 ± 17.4
Test *p*			*F* = 1.361 *p* = 0.259	*F* = 4.397 ***p* = 0.006** **1 > 4** *p = 0.004	*F* = 3.299 ***p* = 0.023** **1 > 4** *p = 0.006	*F* = 3.251 ***p* = 0.025** **1 > 4** *p = 0.009
Treatment cycle	Weekly	15 (13.6)	64.9 ± 10.4	31.6 ± 4.7	26.5 ± 4.9	119.1 ± 16.5
	Every 15 days	38 (34.5)	64.9 ± 10.6	31.1 ± 4.1	23.3 ± 6.6	115.7 ± 17.5
	Every 21 days	54 (49.1)	66.9 ± 11.6	32.4 ± 3.4	25.7 ± 4.9	121.1 ± 14.1
	Every 28 days	3 (2.7)	59.3 ± 7.1	30.0 ± 4.0	21.3 ± 4.5	107.3 ± 9.6
Test *p*			*F* = 0.639 *p* = 0.592	*F* = 1.094 *p* = 0.355	*F* = 2.262 *p* = 0.085	*F* = 1.425 *p* = 0.240

Bold values indicate statistical significance (*p* < 0.05)

^*^Mann–Whitney *U* Bonferroni correction

The mean scores of the SCNS subdimensions and total scores were examined for the patients included in the study, revealing values of 49.3 ± 10.4 for the “faith and spiritual practices” subdimension, 16.8 ± 3.9 for the “to be peaceful and secure until the end of life” subdimension, 21.4 ± 5.0 for the “love and support of relatives” subdimension, 11.9 ± 4.0 for the “being informed about health” subdimension, and 99.4 ± 15.4 for the total score of the SCNS (Table 6).

**Table 6:** Mean SCNS scores of the patients (*n* = 110)

Scale and subdimensions	Min–max	Mean ± SD	Median
Faith and spiritual practices	12–60	49.3 ± 10.4	48.0
To be peaceful and secure until the end of life	4–20	16.8 ± 3.9	16.0
Love and support of relatives	5–25	21.4 ± 5.0	20.0
Informing them about their health	3–15	11.9 ± 4.0	12.0
SCNS total	49–120	99.4 ± 15.4	71.0

*Min–max* Minimum–maximum, *SD* Standard deviation, *SCNS* Spiritual Care Needs Scale

Table 7 shows the comparison of the mean scores of the subdimension and the total scores of the SCNS based on the descriptive characteristics of the patients. It was found that married patients had higher mean scores in the subdimension of love and support of relatives and total scores than single patients (*p* = 0.035, *p* = 0.037, respectively). On the other hand, no statistically significant difference was found between the mean scores of the subdimension and the total scores of the SCNS according to the age, gender, education level, occupation and employment status (*p* > 0.05) (Table 7).

**Table 7:** Comparison of the mean SCNS subdimension and total scores based on the descriptive characteristics of the patients (*n* = 110)

Descriptive characteristics		N (%)	Subdimensions and total score mean scores				
			Faith and spiritual practices X ± SD	To be peaceful and secure until the end of life X ± SD	Love and support of relatives X ± SD	Informing them about their health X ± SD	Total X ± SD
Age	60 ≤	40 (36.4)	48.5 ± 11.6	16.9 ± 4.5	20.8 ± 4.6	12.0 ± 4.0	98.1 ± 16.6
	60 >	70 (63.6)	49.8 ± 9.7	16.7 ± 3.4	21.7 ± 5.3	11.9 ± 4.1	100.1 ± 14.7
Test *p*			*t* = − 0.636 *p* = 0.526	*t* = 0.172 *p* = 0.863	*t* = − 0.964 *p* = 0.337	*t* = 0.143 *p* = 0.887	*t* = − 0.665 *p* = 0.508
Gender	Female	27 (24.5)	49.5 ± 10.3	16.3 ± 4.4	19.8 ± 5.1	13.0 ± 3.3	98.7 ± 15.9
	Male	83 (75.5)	49.3 ± 10.4	16.9 ± 3.7	21.9 ± 4.9	11.6 ± 4.2	99.6 ± 15.3
Test *p*			*t* = 0.099 *p* = 0.921	*t* = − 0.709 *p* = 0.480	*t* = − 1.857 *p* = 0.066	*t* = 1.665 *p* = 0.066	*t* = − 0.281 *p* = 0.779
Marital status	Married	102 (92.7)	49.6 ± 10	16.9 ± 3.8	21.6 ± 4.9	12.1 ± 3.9	100.2 ± 14.7
	Single	8 (7.3)	45.4 ± 14.3	15.1 ± 4.8	17.8 ± 5.9	10.3 ± 5.1	88.5 ± 20.2
Test *p*			*t* = 1.116 *p* = 0.267	*t* = 1.274 *p* = 0.205	*t* = 2.138 ***p* = 0.035**	*t* = 1.228 *p* = 0.222	*t* = 2.116 ***p* = 0.037**
Education level	Illiterate	2 (1.8)	56.0 ± 0.0	20.0 ± 0.0	23.0 ± 2.8	15.0 ± 0.0	114 ± 2.8
	Primary education	69 (62.7)	50.3 ± 8.6	16.8 ± 3.8	22.2 ± 4.4	12.1 ± 3.7	101.4 ± 13
	High school	20 (18.2)	51.2 ± 9.1	17.6 ± 2.9	19.8 ± 5.7	10.8 ± 4.2	99.4 ± 14.5
	University and over	19 (17.3)	42.9 ± 15.2	15.6 ± 4.8	20.0 ± 6.3	12.2 ± 4.9	90.7 ± 21.2
Test *p*			KW = 5.201 *p* = 0.158	KW = 4.228 *p* = 0.238	KW = 5.961 *p* = 0.114	KW = 6.271 *p* = 0.099	KW = 6.327 *p* = 0.097
Occupation	^1^Housewife	17 (15.5)	53.1 ± 6.4	17.4 ± 3.9	21.2 ± 5.0	11.9 ± 3.4	103.5 ± 14.6
	^2^Retired	34 (30.9)	48.7 ± 11.5	17.2 ± 3.5	22.7 ± 5.0	12.6 ± 3.4	101.2 ± 14.6
	^3^Civil servant	19 (17.3)	47.0 ± 12.8	15.9 ± 4.4	21.7 ± 4.9	11.1 ± 5.2	95.8 ± 17.8
	^4^Self-employed	29 (26.4)	51.1 ± 8.4	17.3 ± 3.3	20.4 ± 5.3	11.6 ± 4.5	100.4 ± 14.2
	^5^Worker	11 (10)	44.5 ± 10.4	14.6 ± 4.8	19.5 ± 4.7	12.3 ± 3.7	91 ± 15.7
Test *p*			*F* = 1.665 *p* = 0.164	*F* = 1.448 *p* = 0.223	*F* = 1.252 *p* = 0.294	*F* = 0.462 *p* = 0.763	*F* = 1.563 *p* = 0.190
Employment status	Yes	10 (9.1)	45.0 ± 13.8	15.0 ± 5.1	22.9 ± 4.1	13.5 ± 3.2	96.4 ± 16.3
	No	100 (90.9)	49.7 ± 10.0	17.0 ± 3.7	21.2 ± 5.1	11.8 ± 4.1	99.7 ± 15.3
Test *p*			*t* = − 1.384 *p* = 0.169	*t* = − 1.552 *p* = 0.123	*t* = 1.010 *p* = 0.315	*t* = 1.302 *p* = 0.196	*t* = − 0.644 *p* = 0.521

Bold values indicate statistical significance (*p* < 0.05)

Table 8 provides a comparative analysis of mean scores within subdimensions and total scores of SCNS. A statistically significant increase was observed in patients undergoing chemotherapy in the mean score for the subdimension of belief and spiritual practices compared to those receiving other treatments (*p* = 0.040). On the other hand, no statistically significant difference was found between the mean scores of the subdimension and the total scores of the SCNS according to the diagnosis time, disease condition, history of surgery, history of radiotherapy, number of treatment courses and treatment cycle (*p* > 0.05) (Table 8).

**Table 8:** Comparison of the mean scores of the subdimension and total scores of the SCNS based on the diagnosis and treatment characteristics (n = 110)

Diagnosis and treatment characteristics		N (%)	Subdimensions and total score mean scores				
			Faith and spiritual practices X ± SD	To be peaceful and secure until the end of life X ± SD	Love and support of relatives X ± SD	Informing them about their health X ± SD	Total X ± SD
Diagnosis time	0–1 years	58 (52.7)	48.7 ± 10.9	16.9 ± 3.7	21.4 ± 5.0	11.8 ± 4.0	98.8 ± 16.7
	1–3 years	30 (27.3)	48.4 ± 11.3	16.2 ± 4.5	20.8 ± 6.0	12.2 ± 4.5	97.6 ± 16.1
	3 years and over	22 (20)	52.3 ± 6.8	17.2 ± 3.4	21.9 ± 3.9	11.9 ± 3.5	103.2 ± 9.3
Test *p*			*F* = 1.132 *p* = 0.326	*F* = 0.510 *p* = 0.602	*F* = 0.320 *p* = 0.727	*F* = 0.120 *p* = 0.887	*F* = 0.918 *p* = 0.402
Disease condition	Primary	72 (65.5)	49.6 ± 9.8	16.9 ± 3.5	21.1 ± 5.6	11.5 ± 4.4	99.0 ± 14.7
	Metastatic	38 (34.5)	48.8 ± 11.5	16.6 ± 4.5	21.9 ± 3.7	12.8 ± 3.1	100.1 ± 16.7
Test *p*			*t* = 0.361 *p* = 0.719	*t* = 0.314 *p* = 0.754	*t* = − 0.761 *p* = 0.448	*t* = − 1.647 *p* = 0.102	*t* = − 0.353 *p* = 0.725
History of surgery	Yes	43 (39.1)	50.4 ± 11.1	17.1 ± 4.4	22.2 ± 3.9	12.1 ± 3.8	101.7 ± 15.9
	No	67 (60.9)	48.6 ± 9.9	16.6 ± 3.5	20.9 ± 5.6	11.8 ± 4.2	97.9 ± 14.9
Test *p*			*t* = − 0.898 *p* = 0.371	*t* = − 0.607 *p* = 0.545	*t* = − 1.335 *p* = 0.185	*t* = − 0.345 *p* = 0.731	*t* = − 1.292 *p* = 0.199
History of radiotherapy	Yes	72 (65.5)	49.5 ± 9.6	16.6 ± 3.7	21.2 ± 5.1	11.8 ± 4.1	99.1 ± 13.6
	No	38 (34.5)	49 ± 11.8	17.1 ± 4.1	21.6 ± 5.0	12.2 ± 3.9	99.9 ± 18.4
Test *p*			*t* = − 0.207 *p* = 0.837	*t* = 0.568 *p* = 0.571	*t* = 0.403 *p* = 0.688	*t* = 0.485 *p* = 0.628	*t* = 0.262 *p* = 0.794
Current treatment	Chemotherapy	72 (65.5)	50.8 ± 9.4	17.2 ± 3.6	21.5 ± 5.2	11.6 ± 4.1	101.1 ± 14.7
	Other (Chemoradiotherapy or target therapy)	38 (34.5)	46.5 ± 11.7	15.9 ± 4.3	21.2 ± 4.8	12.6 ± 3.8	96.2 ± 16.2
Test *p*			*t* = 2.075 ***p* = 0.040**	*t* = 1.683 *p* = 0.095	*t* = 0.309 *p* = 0.758	*t* = − 1.188 *p* = 0.238	*t* = 1.603 *p* = 0.112
Number of treatment courses	0–5. courses	31 (28.2)	49.6 ± 12.6	17.8 ± 3.4	21.0 ± 5.4	12.7 ± 3.0	101.2 ± 19.6
	6–10. courses	23 (20.9)	51.8 ± 7.7	17.7 ± 3.1	21.0 ± 4.6	10.7 ± 4.7	101.2 ± 13.1
	11–20. courses	24 (21.8)	48.8 ± 8.4	15.8 ± 4.1	22.0 ± 4.9	10.3 ± 5.1	96.8 ± 12.9
	More than 20 courses	32 (29.1)	47.6 ± 11	15.9 ± 4.3	21.5 ± 5.3	13.3 ± 2.8	98.3 ± 14.2
Test *p*			KW = 3.418 *p* = 0.332	KW = 6.716 *p* = 0.082	KW = 2.203 *p* = 0.531	KW = 7.346 *p* = 0.062	KW = 5.192 *p* = 0.158
Treatment cycle	Weekly	15 (13.6)	50.5 ± 6.3	16.9 ± 3.0	22.5 ± 3.3	13.3 ± 2.7	103.2 ± 8.9
	Every 15 days	38 (34.5)	47.9 ± 10.9	16.9 ± 3.4	21.4 ± 4.3	12.2 ± 3.8	98.5 ± 13.5
	Every 21 days	54 (49.1)	50.1 ± 11.2	17.1 ± 4.0	21.1 ± 6	11.4 ± 4.5	99.6 ± 17.9
	Every 28 days	3 (2.7)	46 ± 3.6	10.3 ± 5.7	20 ± 4	11 ± 1.7	87.3 ± 9.3
Test *p*			KW = 3.758 *p* = 0.289	KW = 6.260 *p* = 0.100	KW = 1.871 *p* = 0.600	KW = 3.640 *p* = 0.303	*F* = 0.970 *p* = 0.410

Bold values indicate statistical significance (*p* < 0.05)

## Discussion

This study aimed to assess the spiritual well-being levels and spiritual care needs of individuals diagnosed with lung cancer. The findings revealed that the patients exhibited high spiritual well-being levels, with the “harmony with nature” subdimension scoring particularly high in relation to spiritual well-being. This aligns with several studies which reported high spiritual well-being of cancer patients (Martins et al., 2020; Rabow & Knish, 2015).

In the current study, no significant differences were found in the mean scores of the TFSWBS subdimensions and total scores concerning the age, gender, and marital status of the patients. Consistent with similar studies involving lung cancer or other cancer types, this study aligns with the absence of significant differences in age, gender, and marital status among patients (Gudenkauf et al., 2019; Kahraman & Pehlivan, 2023; Martins et al., 2020; Rabow & Knish, 2015). In contrast to this study, previous research has indicated that older, female, and married cancer patients tend to exhibit higher levels of spiritual well-being (Frost et al., 2013; Gudenkauf et al., 2019; Kamijo & Miyamura, 2020; Martins et al., 2020; Munoz et al., 2015; Piderman et al., 2015).

In our study, participants with a university degree or higher displayed higher mean scores in the harmony with nature and anomie subdimensions of spiritual well-being. This aligns with another study, which reported a connection between the spiritual well-being of cancer patients and their level of education (Riklikiene et al., 2020). However, unlike our findings, some studies have reported no significant influence of patients’ educational status on their spiritual well-being levels (Kahraman & Pehlivan, 2023; Martins et al., 2020). Regarding employment status, our study did not reveal a significant difference in overall spiritual well-being among patients. Nevertheless, civil servants demonstrated higher mean scores in the harmony with nature subdimension than workers, contrasting with Martins et al.’s (2020) study that found no significant difference between occupation and spiritual well-being (Martins et al., 2020). It is thought in our study that the higher mean scores in the harmony with nature subdimension among civil servants may be linked to their higher level of education.

Our study identified higher mean scores in the anomie subdimension of spiritual well-being in patients diagnosed between 0–1 years compared to those with a diagnosis within 3 years and more. In contrast, another study found no significant difference in the spiritual well-being scale scores based on the time of diagnosis (Çınar & Şirin, 2019). In our study, there was no significant difference observed in the mean subdimension and total scores of the TFSWBS based on disease status, history of surgery, or radiotherapy. This aligns with similar studies where no significant differences were reported between disease stage and spiritual well-being (Piderman et al., 2015; Rabow & Knish, 2015). However, it is important to note that the results in the literature vary. In one study, patients with metastasis had higher mean scores in the anomie subdimension, while patients without metastasis showed higher mean scores in transcendence and total spiritual well-being (Çınar & Şirin, 2019). Another study found that patients without metastasis exhibited higher spiritual well-being (Jetan et al., 2023).

Additionally, a study reported that early stage disease had a negative impact on spiritual well-being (Kamijo & Miyamura, 2020). Given the significant role of chemotherapy in lung cancer treatment, our study revealed that patients undergoing chemotherapy had higher mean scores in the transcendence subdimension than those receiving other treatments. This differs from another study where cancer patients who underwent surgical treatment showed higher levels of spiritual well-being (Zübeyde & Özlem, 2023). These discrepancies in study outcomes may be attributed to variations in patient satisfaction levels with the healthcare system. The symptom burden imposed by lung cancer, coupled with a poor prognosis and prolonged treatment, can significantly impact both the physical and spiritual well-being of patients (Lehto, 2017). In our study, we found that the number of treatment cycles had an impact on spiritual well-being, while the specific treatment cycle itself did not show a significant effect. Notably, individuals with 5 or fewer treatment cycles exhibited higher mean scores in the harmony with nature and anomie subdimensions and total spiritual well-being. This suggests that as the number of treatment cycles increases, spiritual well-being tends to decline. These findings suggest that patients undergoing long-term treatment may experience a diminishing belief in recovery, life expectations, and hope.

The study identified high spiritual needs among the patients, with the “informing them about their health” subdimension obtaining the highest scores. Notably, in the current study, it was observed that patients’ needs in the informing them about their health increased, deviating from the emphasis on love, support, and bonding seen in other studies (Kırca et al., 2023; Shi et al., 2023). This divergence is thought to be linked to differences in the disease stage.

In our study, there was no significant difference in the mean scores of subdimensions and total scores of the spiritual care needs scale based on age, gender, educational status, occupation, and employment status of the patients. This is consistent with several other studies reporting no significant association between the age and gender of cancer patients and their spiritual needs (Bussing et al., 2013; Forouzi et al., 2017). However, a study with lung cancer patients (2021) found a negative association between age and religious needs as well as inner peace needs (Fradelos et al., 2021). Forouzi et al. (2017) discovered that older patients and those with higher education levels were more likely to experience spiritual needs in a study involving patients with various cancers (Forouzi et al., 2017). Similarly, research on individuals with chronic diseases reported higher religious needs among the elderly and women (Bussing et al., 2015). Another study focusing on patients with chronic pain and cancer found that women had high existential, religious, and inner peace needs compared to men, and those with a high school education exhibited higher inner peace needs (Bussing et al., 2013). In contrast, Kamijo and Miyamura (2020) reported that younger cancer patients required additional assistance to meet their spiritual needs in a study with patients facing various cancers (Kamijo & Miyamura, 2020). Moreover, Kırca et al. (2023) found that the spiritual care needs of cancer patients increased with undergraduate/graduate education levels (Kırca et al., 2023). It is suggested that differences in population characteristics and education systems between countries may contribute to variations in spiritual needs.

Family members play a crucial role in addressing the spiritual needs of individuals diagnosed with cancer (Hatamipour et al., 2015). Married patients, in particular, were found to express a greater need for love and support from their relatives, indicating higher overall spiritual care needs. The findings of another study conducted in Turkey, similar to the present study, reported an increase in the spiritual care needs of married patients (Kırca et al., 2023). However, in contrast to our results, other studies indicated that patients living alone, as well as single, widowed, or divorced patients, exhibited higher spiritual needs (Bussing et al., 2013; Shi et al., 2023). These disparities suggest that family members in Turkey may face challenges in showing sufficient interest or providing social support to cancer patients, potentially due to difficulties in the care and treatment of cancer.

Furthermore, a significant difference was observed in the spiritual care needs of patients based on their current treatment. Specifically, patients receiving only chemotherapy (CT) had higher mean scores for belief and spiritual practices than those receiving other treatments. This implies that the increase in spiritual care needs during CT treatment may be attributed to patients experiencing high psychosocial issues such as sadness, anxiety, fear, and decreased coping mechanisms. Meeting these spiritual care needs becomes crucial to addressing the psychosocial challenges associated with cancer treatment. Nurses, being integral healthcare providers, hold a key role in addressing and fulfilling these spiritual care needs.

### Limitations

It is acknowledged that the research was conducted in a single center, limiting the generalizability of the results to lung cancer patients receiving outpatient treatment in that specific hospital. Additionally, the study recognized that spiritual needs are closely related to the severity of cancer. The absence of an evaluation of spiritual needs according to the stages of the disease represents another notable limitation in this study.

## Conclusion

In conclusion, the study revealed that lung cancer patients exhibited high levels of both spiritual well-being and spiritual care needs. Specifically, patients had higher mean scores in the “harmony with nature” subdimension of spiritual well-being, while the subdimensions of “informing them about their health,” “to be peaceful and secure until the end of life” and “love and support of relatives” had higher scores in terms of spiritual care needs, respectively. Certain demographic and treatment-related factors were associated with variations in spiritual well-being and needs. Patients with a university degree or higher, civil servant or self-employed, those receiving only CT, and those receiving 5 cycles or less of CT exhibited higher levels of spiritual well-being. On the other hand, the spiritual care needs of married patients and patients receiving only CT were found to be higher.

Recognizing the significance of spirituality as part of holistic care, health personnel, particularly nurses, play a crucial role in understanding and addressing the spiritual well-being levels and needs of cancer patients. Nurses should be able to openly express their views on spirituality, support patients to share their spiritual needs with them, and have the knowledge, skills and experience to assess or respond to patients' spiritual needs. Overall, this study will contribute to nurses’ understanding of patients' spiritual feelings, such as hope, peace, and love, and meeting their spiritual needs alongside medical care interventions.
