# Correction to: Evaluation of Spiritual Care and Well-Being Levels of Individuals Diagnosed with Lung Cancer in Turkey

**DOI:** 10.1007/s10943-024-02142-0

**Published:** 2024-10-08

**Authors:** Seher Çakmak, Melike Demir Doğan, Nisanur Selim, Gülse Nur Kalleci

**Affiliations:** 1 Department of Internal Medicine Nursing, Faculty of Health Sciences, Gümüşhane University, Gümüşhanevî Campus, Bağlarbaşı Street, 29100 Gümüşhane, Turkey; 2 Department of Nursing, Faculty of Health Sciences, Gümüşhane University, Gümüşhanevî Campus, Bağlarbaşı Street, 29100 Gümüşhane, Turkey

**Correction to: Journal of Religion and Health** 10.1007/s10943-024-02088-3

In this article, the 1st and 6th paragraphs in the “**Discussion**” section contain several errors.

The corrected paragraphs are given below.

## Discussion

This study aimed to assess the spiritual well-being levels and spiritual care needs of individuals diagnosed with lung cancer. The findings revealed that the patients exhibited high spiritual well-being levels, with the “harmony with nature” sub dimension scoring particularly high in relation to spiritual well-being. This aligns with several studies which reported high spiritual well-being of cancer patients (Martins et al., 2020; Rabow & Knish, 2015).”

The study identified high spiritual needs among the patients, with the specific subdimension “informing them about their health” obtaining the highest scores. Notably, in the current study, it was observed that patients’ needs in terms of “informing them about their health” increased, deviating from the emphasis on love, support, and bonding seen in other studies (Kırca et al., 2023; Shi et al., 2023). This divergence is thought to be linked to differences in the disease stage.

The original article has been corrected.
